# Long‐Term Survival After Desperation Surgery for Primary Mediastinal Non‐Seminomatous Germ Cell Tumor With Rising Alpha‐Fetoprotein

**DOI:** 10.1002/iju5.70151

**Published:** 2026-03-07

**Authors:** Tomohiko Aigase, Takeshi Kishida, Hayato Kubo, Takahiro Matsumoto, Atsuto Suzuki, Kota Washimi, Yoichiro Okubo, Satoshi Hara, Hitoshi Sugiura, Noboru Nakaigawa

**Affiliations:** ^1^ Department of Urology Kanagawa Cancer Center Yokohama Kanagawa Japan; ^2^ Department of Pathology Kanagawa Cancer Center Yokohama Kanagawa Japan; ^3^ Department of Urology Kawasaki Municipal Hospital Kawasaki Kanagawa Japan; ^4^ Department of Pathology Kawasaki Municipal Hospital Kawasaki Kanagawa Japan

**Keywords:** alpha‐fetoprotein, desperation surgery, germ cell tumor, mediastinal tumor, tumor markers

## Abstract

**Introduction:**

Primary mediastinal non‐seminomatous germ cell tumors (PMNSGCTs) are rare and aggressive neoplasms that carry a poor prognosis, especially following salvage chemotherapy. In cases of residual tumors with elevated tumor marker levels, desperation surgery may be considered.

**Case Presentation:**

A 32‐year‐old man with PMNSGCT presented with a 100‐mm anterior mediastinal mass and elevated alpha‐fetoprotein (AFP; 17 000 ng/mL). After induction and salvage chemotherapy, AFP levels decreased to 20 ng/mL but did not normalize, and a 55‐mm cystic tumor persisted. Desperation surgery was performed despite an increase in AFP (40 ng/mL) before the operation. The tumor was completely resected, and histopathological examination revealed no viable tumor cells or teratomas. Postoperatively, AFP normalized, and the patient has remained recurrence‐free.

**Conclusion:**

This case highlights the potential role of desperation surgery in achieving long‐term survival in selected patients with PMNSGCT, despite increased levels of tumor markers.

## Keynote Message

Desperation surgery may facilitate long‐term survival in selected patients with primary mediastinal non‐seminomatous germ cell tumors, despite elevated tumor marker levels after chemotherapy. Careful patient selection and complete resection are essential.

## 1 Introduction

Primary mediastinal germ cell tumors (PMGCTs) are rare neoplasms [1]. According to the International Germ Cell Consensus Classification, mediastinal primary tumors are categorized as having a poor prognosis [2]. Even with standard treatment, the 5‐year overall survival rate is only 40%–50%, likely due to their intrinsic chemoresistance and aggressive histological features [3, 4].

The standard treatment for PMGCTs comprises platinum‐based combination chemotherapy, followed by surgical resection of residual masses once tumor markers normalize [2, 5].

Despite the suggestive presence of active residual cancer, surgical resection may be considered in cases where tumor markers remain elevated despite aggressive chemotherapy. Such a surgery is termed “desperation surgery.” [6] Its indications remain controversial, and are generally limited to select patients with resectable disease when no other effective options are available [7]. Although high‐level evidence is lacking, recent guidelines have proposed potential benefits in carefully selected cases [8, 9].

Herein, we report a rare case of a patient with a primary mediastinal non‐seminomatous germ cell tumor (PMNSGCT) who exhibited persistently abnormal and rising tumor marker levels after multiple courses of chemotherapy and achieved long‐term recurrence‐free survival following desperation surgery without further treatment.

## 2 Case Presentation

**FIGURE 1:**
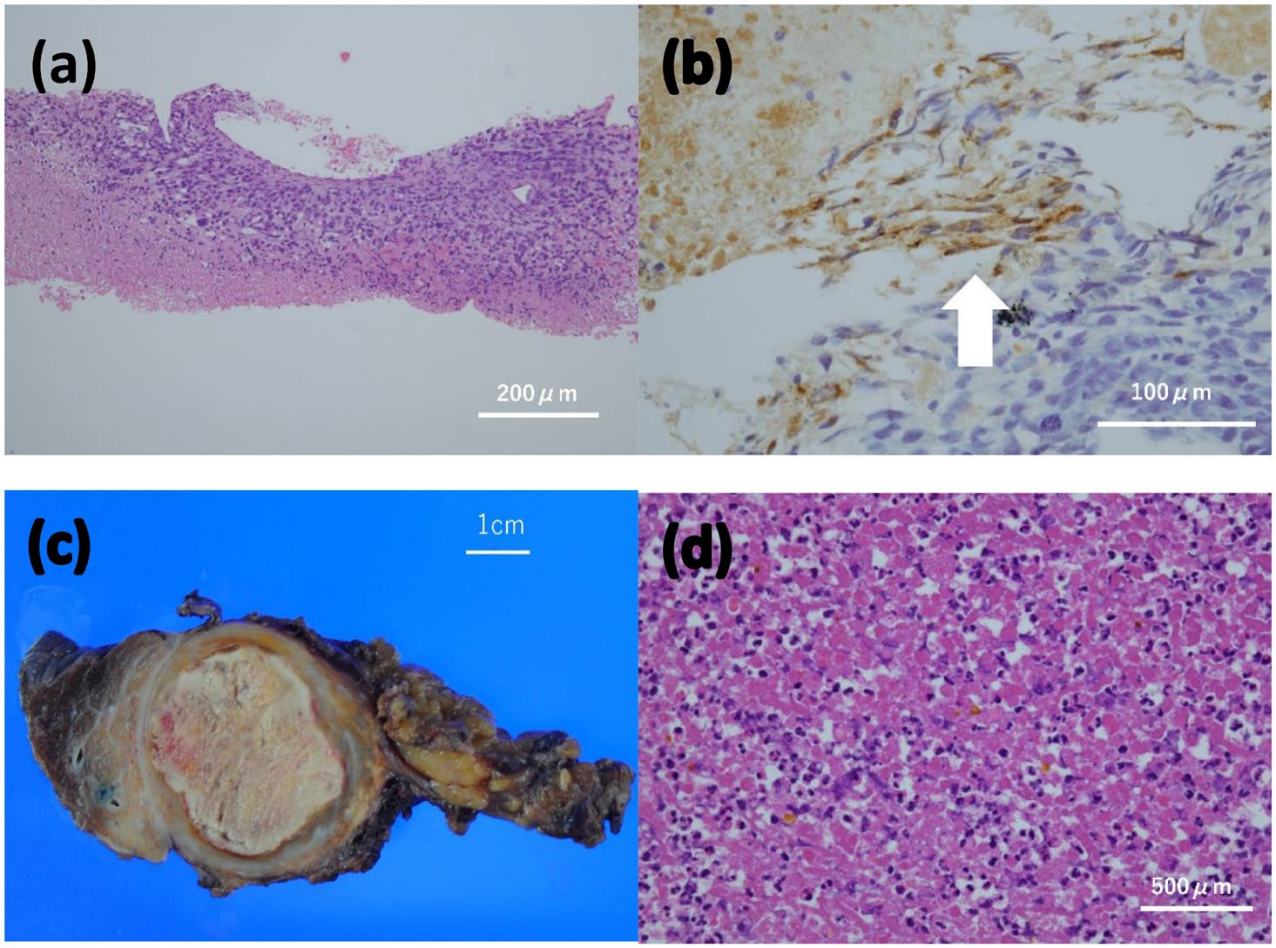
Histopathological findings. (a) Hematoxylin and eosin (H&E) staining of the CT‐guided biopsy specimen (original magnification ×100) showing proliferation of tumor cells with nuclear enlargement, suggestive of malignancy. (b) Immunohistochemical staining for alpha‐fetoprotein (AFP) using an anti‐AFP antibody (×400), demonstrating focal AFP positivity indicated by arrows. (c) Gross specimen of the resected mediastinal tumor, revealing a yellow‐white solid mass with internal necrosis. (d) H&E staining of the resected specimen (×400) showing no viable tumor cells.

**FIGURE 2:**
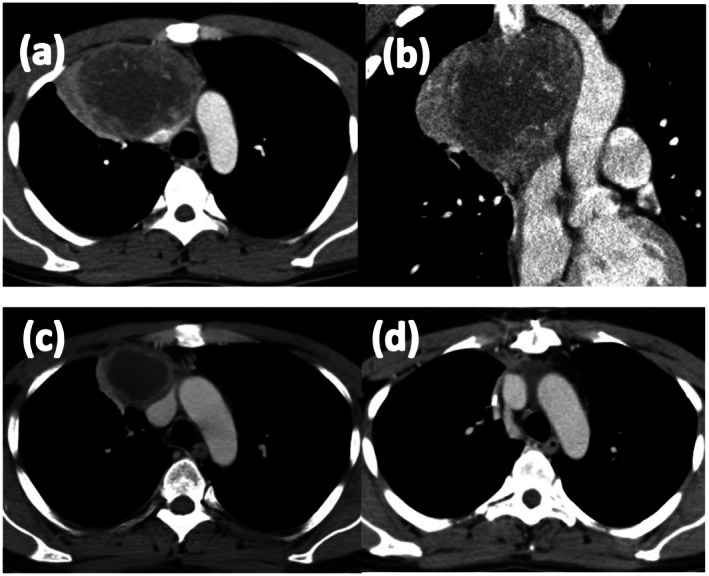
Contrast‐enhanced computed tomography (CT) images of the anterior mediastinal tumor. (a) Axial view at initial diagnosis reveals a 100‐mm solid mass in the anterior mediastinum. (b) Coronal view of the same lesion confirms its extent with no evidence of distant metastasis. (c) Axial view just before surgery shows a residual 55‐mm cystic mass. (d) Axial view after surgery demonstrates complete resection with no evidence of residual tumor.

A 32‐year‐old man presented with a fever. Chest computed tomography (CT) at the referral hospital revealed an anterior mediastinal mass. CT‐guided biopsy indicated tumor cells with enlarged nuclei (Figure 1a). Immunohistochemically, the tumor was focally positive for alpha‐fetoprotein (AFP) (Figure 1b), glypican‐3, and c‐KIT. Considering these findings, a yolk sac tumor was strongly suspected. The patient was referred to our institution for further treatment. Laboratory evaluation revealed the following results: AFP, 17 000 ng/mL; AFP‐lectin 3 fraction > 99.6%; and human chorionic gonadotropin (HCG) < 1.0 IU/mL. No abnormalities were observed. Contrast‐enhanced CT revealed a 100‐mm solid mass in the anterior mediastinum with enhancement (Figure 2a,b), and no distant metastases were identified. Scrotal ultrasonography revealed no abnormality. Based on these findings, the patient was diagnosed with PMNSGCT, and systemic chemotherapy was initiated.

**FIGURE 3:**
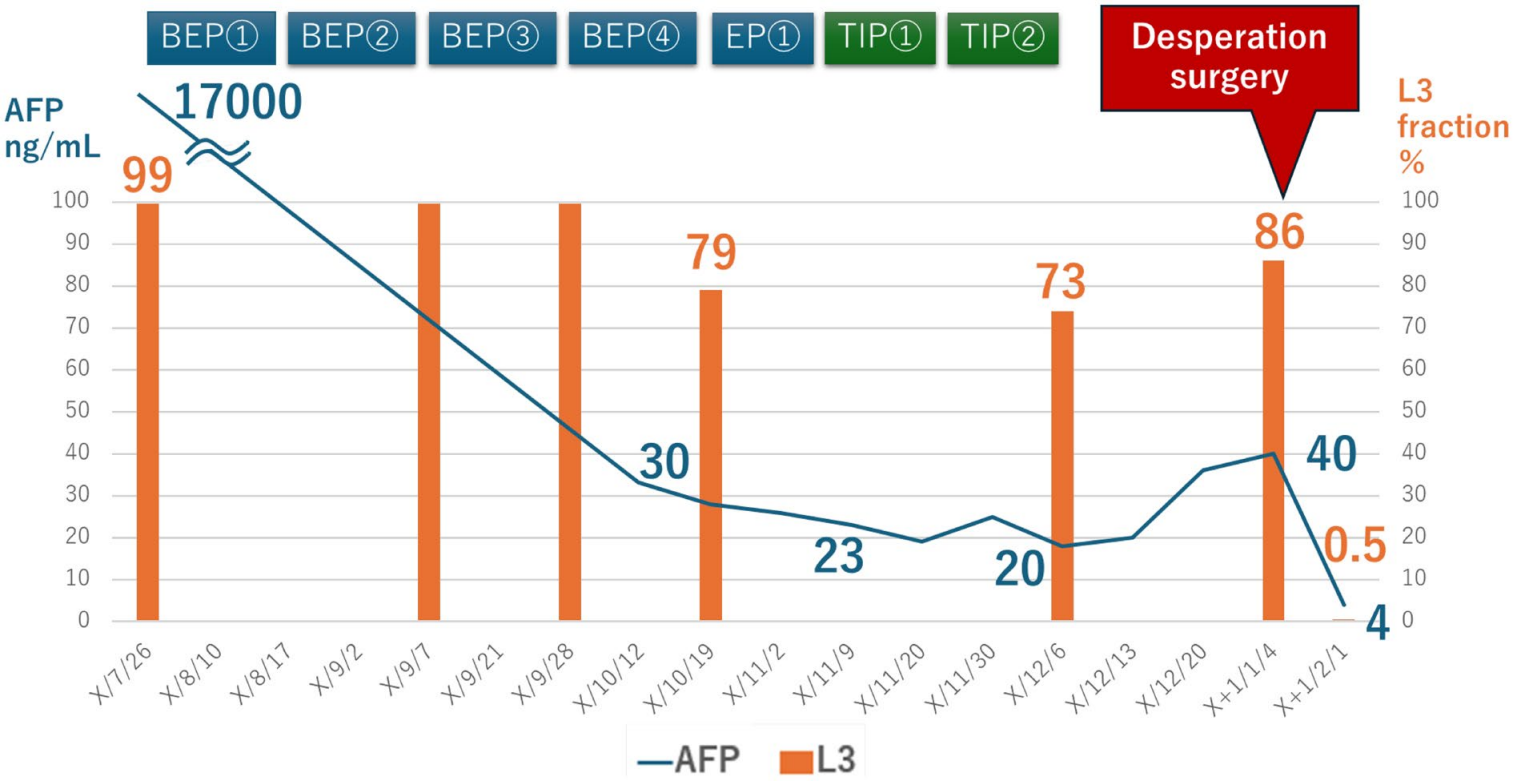
Timeline of chemotherapy administration and tumor marker kinetics. Changes in serum alpha‐fetoprotein (AFP) and AFP‐L3 fraction levels are plotted in relation to the timing of BEP, TIP, and EP chemotherapy regimens. BEP: Bleomycin, etoposide, and cisplatin; TIP: Paclitaxel, ifosfamide, and cisplatin; EP: Etoposide and cisplatin.

The treatment course is summarized in Figure 3. The patient received four cycles of bleomycin, etoposide, and cisplatin as first‐line chemotherapy. AFP subsequently decreased to 30 ng/mL but did not normalize. Imaging revealed a decreased residual mass with cystic changes. One cycle of etoposide and cisplatin was administered as additional therapy, but the AFP response was poor (23 ng/mL). Two cycles of salvage chemotherapy with paclitaxel, ifosfamide, and cisplatin (TIP) resulted in a modest decrease in the AFP levels to 20 ng/mL. CT revealed a 55‐mm residual tumor with cystic changes in the anterior mediastinum (Figure 2c). Brain magnetic resonance imaging was not performed at that time because the patient had no neurological symptoms, and no clinical findings suggested central nervous system involvement.

Given the persistently elevated tumor marker levels and residual tumor on imaging, surgery was planned. Although AFP increased up to 40 ng/mL just before the operation, the patient underwent surgical resection 1 month after the final TIP course. The tumor was completely resected, and histopathological examination revealed no residual tumor or teratoma elements (Figure 1c,d). The AFP level normalized to 4 ng/mL postoperatively, and CT confirmed complete resection (Figure 2d). The patient is being followed up with serial AFP measurements and imaging and remains recurrence‐free without additional treatment for 7 years postoperatively.

## 3 Discussion

Previous studies have reported poor survival rates in patients with mediastinal tumors undergoing salvage chemotherapy [1]. Compared to retroperitoneal germ cell tumors, PMNSGCT respond poorly to salvage regimens, and no specific regimen has demonstrated superior outcomes [10, 11, 12].

In patients with PMNSGCT, elevated serum marker levels after chemotherapy do not always correlate with residual viable disease. Approximately 30%–40% of patients undergoing desperation surgery have no residual cancer, with pathology demonstrating only teratoma or necrotic tissue [13]. This discordance may be explained by the leakage of AFP or HCG from the accumulated fluid of the cystic components rather than actual tumor activity [14, 15]. Although leakage of AFP into the cystic fluid likely contributed to the persistent serum AFP elevation in this case, AFP levels in the cystic fluid were not measured, which is a limitation of this report.

In the present case, AFP levels reached a plateau following TIP salvage chemotherapy, indicating a limited likelihood of further response to additional systemic therapy. According to the National Comprehensive Cancer Network (NCCN) and European Association of Urology guidelines, surgical resection should be considered when serum tumor markers plateau and the residual mass is surgically resectable [8, 9]. Imaging findings in our case also demonstrated a cystic component, raising the possibility of AFP accumulation rather than a viable tumor. Furthermore, third‐line chemotherapy, including irinotecan‐based regimens, provides limited benefit in PMNSGCT and is generally regarded as palliative rather than curative. These considerations supported the decision to proceed with desperation surgery.

Based on these factors some reports recommend surgical resection of resectable mediastinal masses regardless of post‐chemotherapy tumor marker levels [13, 16, 17]. Although desperation surgery in PMNSGCT generally shows poor outcomes, with reported 5‐year survival rates of 25%–35% and median overall survival of 9.7–11.5 months [13, 18], long‐term survival is achieved in some cases. Furthermore, patients with elevated AFP at the time of residual tumor resection reportedly have more favorable outcomes than those with elevated HCG. Ong et al. reported significantly better survival among patients with AFP elevation compared with HCG elevation in the setting of post‐chemotherapy mediastinal germ cell tumors [19]. This prognostic distinction may help contextualize the favorable long‐term course observed in the present case despite persistent AFP elevation. Reported favorable prognostic factors include localization of the disease to the mediastinum, achievement of complete resection, normalization of tumor markers postoperatively, and the absence of viable tumor on pathological examination [16, 18, 20]; the present case fulfilled these criteria. In contrast to these favorable prognostic factors, the NCCN guidelines recommend proceeding with desperation surgery only when tumor markers are mildly elevated and trending downward. For patients with persistently elevated markers, as in our case, further chemotherapy is generally advised.

This case is unique in that the decision to proceed with surgery was made despite rising AFP levels after salvage chemotherapy—an indication in which further systemic therapy is typically recommended. The combination of plateauing AFP levels and a predominantly cystic residual mass suggested limited viable tumor, allowing multidisciplinary judgment to favor resection. This case therefore demonstrates that carefully selected patients may still achieve excellent long‐term outcomes and help avoid unnecessary administration of salvage chemotherapy with timely desperation surgery, even in the presence of increasing tumor markers.

## Funding

The authors have nothing to report.

## Ethics Statement

The authors have nothing to report.

## Consent

Written informed consent was obtained from the patient for publication of this case report and any accompanying images.

## Conflicts of Interest

The authors declare no conflicts of interest.

## Data Availability

The data that support the findings of this study are available on request from the corresponding author. The data are not publicly available due to privacy or ethical restrictions.
